# Mapping of transcription start sites of human retina expressed genes

**DOI:** 10.1186/1471-2164-8-42

**Published:** 2007-02-07

**Authors:** Valeria Roni, Ronald Carpio, Bernd Wissinger

**Affiliations:** 1 Molecular Genetics Laboratory, University Eye Hospital, Roentgenweg 11, 72076 Tuebingen, Germany.

## Abstract

**Background:**

The proper assembly of the transcriptional initiation machinery is a key regulatory step in the execution of the correct program of mRNA synthesis. The use of alternative transcription start sites (TSSs) provides a mechanism for cell and tissue specific gene regulation. Our knowledge of transcriptional initiation sequences in the human genome is limited despite the availability of the complete genome sequence. While genome wide experimental and bioinformatic approaches are improving our knowledge of TSSs, they lack information concerning genes expressed in a restricted manner or at very low levels, such as tissue specific genes.

**Results:**

In this study we describe the mapping of TSSs of genes expressed in human retina. Genes have been selected on the basis of their physiological or developmental role in this tissue. Our work combines *in silico *analysis of ESTs and known algorithm predictions together with their experimental validation via Cap-finder RACE. We report here the TSSs mapping of 54 retina expressed genes: we retrieved new sequences for 41 genes, some of which contain un-annotated exons. Results can be grouped into five categories, compared to the RefSeq; (i) TSS located in new first exons, (ii) splicing variation of the second exon, (iii) extension of the annotated first exon, (iv) shortening of the annotated first exon, (v) confirmation of previously annotated TSS.

**Conclusion:**

*In silico *and experimental analysis of the transcripts proved to be essential for the ultimate mapping of TSSs. Our results highlight the necessity of a tissue specific approach to complete the existing gene annotation. The new TSSs and transcribed sequences are essential for further exploration of the promoter and other *cis-*regulatory sequences at the 5'end of genes.

## Background

The spatial and temporal regulation of gene transcription is primarily determined by it's flanking promoter (*cis-*regulatory DNA elements) through interaction with *trans*-acting regulatory proteins (transcription factors) [1,2]. The start of transcription is accomplished by the formation of a pre-initiation complex on the DNA, yet our knowledge of transcriptional initiation sequences in the human genome is still limited despite the availability of the complete genome sequence [3,4]. Therefore one of the main remaining challenges is to locate these gene sequences, defined as the transcription start site (TSS), in order to explore core promoter and *cis-*regulatory elements that direct the start of every transcript. Genomic structure and full length cDNA sequences aligned on the genome provide opportunities to locate TSSs. Conventional methods for determining exact TSSs, such as 5' RACE or primer extension are laborious and are not selective for the complete transcript. Consequently, many mRNA sequences stored in public databases lack information about their genuine 5' ends, mainly due to the difficulties in obtaining full-length cDNA. Several bioinformatic and experimental approaches have been developed to explore full-length cDNAs and the human transcriptome [5]. Computational predictions may represent a powerful tool to localize first exons and TSSs on an averaged genome-wide scale [6,7], however they may fail at the level of individual genes or in genes with complex regulatory patterns (e.g. multiple or tissue-specific TSS).

Recently a number of experimental approaches to compile TSSs on a genome-wide scale have been established including the Database of human Transcriptional Start Sites (DBTSS) [8], whole genome tilling array analysis [9], and the exploration of mouse and human CAGE tag libraries [10]. To enable future progress we need to complete and revise these catalogues with an accurate annotation of the 5' and 3'end, and include splice isoforms of the transcripts. In addition to genome wide approaches, there is a need for more specific studies, which cover tissue specific genes, expressed in a restricted manner. Identification of potential transcription signals that are tissue specific relies on the correct determination of transcriptional start sites.

In this work we describe an experimental approach to identify the TSSs of a selected group of genes, which are predominantly expressed in retina. We focused our attention on the human retina, due to its unique and specialised function. This complex tissue, composed of multiple, highly differentiated and specific cell types (e.g. rod and cone photoreceptors, amacrine cells, Mueller glial cells), expresses a large number of specific genes. Mutations in many of these genes result in blinding disorders. The subset of genes expressed in human retina has been partially elucidated [11,12], with a number of studies defining genes that are either highly expressed in retina or which pose a crucial target of transcription factors in this tissue [13-16]. We selected a pool of retina expressed genes and employed Cap selective RACE to ensure amplification and subsequent cloning of genuine TSSs. We describe herein the results of this analysis, reporting the correct TSSs within this group of retinal transcripts.

## Results

### Genes Selection and *in silico *assembly

76 annotated genes were selected for analysis. The selection was done based on the following criteria: (i) specific or high levels of expression in retina, (ii) a role in retina specific physiological processes or retinal development, and (iii) involvement in retinal disease. A compilation of all tested genes including gene symbol, definition, chromosomal location and tissue/cell type of expression is shown in Additional file 1.

cDNA and transcript sequences available in public databases (RefSeq, NCBI and Ensemble gene predictions covered by at least one EST, Unigene ESTs database) were downloaded and new assemblies generated using SeqMan.

We found that 5' transcript termini represented in public datasets can be readily identified by clusters of cDNA ends in the assemblies. Additionally, the information about putative TSSs was assessed in The Eukaryotic Promoter Database [17] and Database of Transcriptional Start Sites (DBTSS) [8]. These data were compiled to create a preliminary gene model which was used to design primers for the subsequent Cap-finder RACE experiments.

### Experimental examination of TSSs

Cap-finder RACE cDNA fragments were cloned and a variable number of clones were sequenced for each gene, depending on the number and the sizes of the colony PCR products detected on the gel. We obtained products for 54 genes out of the 76 genes analysed. A summary of the results obtained with Cap-finder RACE is shown in Tables 1. Genes for which the promoter and TSS were already known (*RHO *and *OPN1SW*) served as internal positive controls. For each gene we detected at least one splice variant that agreed with one or more RefSeq annotated exons.

Our strategy relies on the location of gene specific primers within internal exons. We obtained those cDNA products that covered at least one exon-exon junction and thus ruled out the possibility of amplification of genomic contamination. This strategy has enabled us to identify alternatively spliced 5' ends that arise from tissue specific gene expression and regulation.

Table 1 lists the results of the Cap-finder RACE experiments for 54 retinal expressed genes and the corresponding RefSeq entry (database release 18). These results can be grouped into five categories with reference to RefSeq; (i) new TSSs within novel exons (8 genes), (ii) alternative splice form of the second exon (2 genes: *IMPG1, SAG*), (iii) extension of the annotated first exon (27 genes), (iv) length shortening of the annotated first exon (4 genes), (v) confirmation of previously annotated TSSs (13 genes). Table 1 provides the exact nucleotide positions of 5' termini of the Cap-finder RACE cDNA clones referring to the UCSC Human Genome Browser (March 2006 assembly). In defining the interval where TSSs are located we report the start, and when present, the internal frequent start and end nucleotide position of each TSS. Sequences from this study have been submitted to GenBank under the accession numbers: DQ067456–DQ067464, DQ426859–DQ426897, DQ980599–DQ980621.

### Retinal expressed genes with new 5' exons

For 8 genes (*C1orf32, CNGA3, DHRS3, ELOVL5, KIFC3, RCV1, RDH12, SLC24A2*) we have identified a new exon composition at the 5' end of the transcript and in some cases new untranslated 5' exons that locate the TSS several kilobases upstream or downstream from the annotated one.

**Figure 1:**
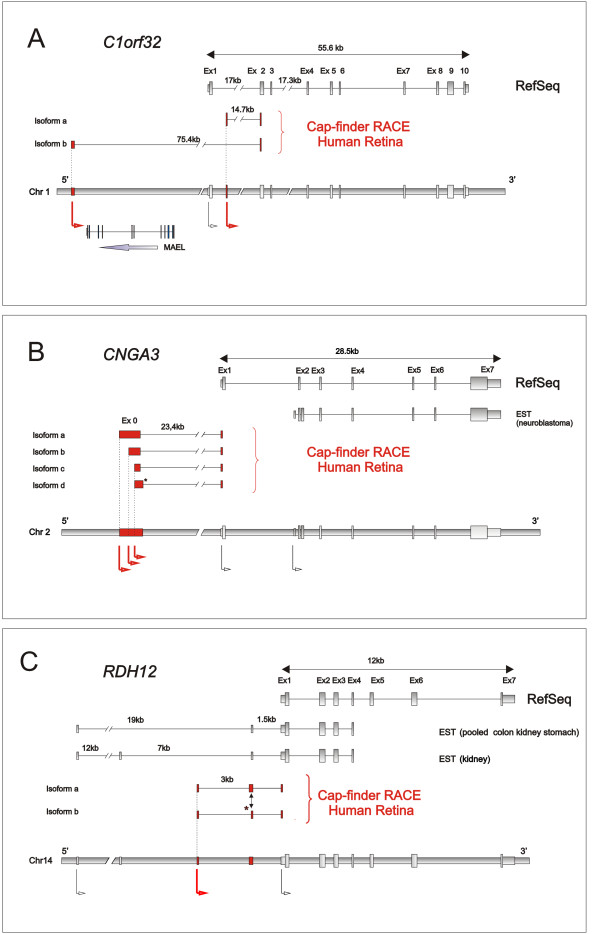
**Schematic gene structure of 3 analysed genes**. Schema of the RefSeq, ESTs, exonic structure of new isoforms identified with the Cap-finder RACE of human retina mRNA and the genomic structure containing the TSSs indicated by arrows. Red Arrows indicate new retina TSSs. A) *C1orf32*. Transcripts in human retina: isoform a contains a new first exon located 2.7 kb downstream from previous TSS, isoform b presents a first exon 58,5 kb upstream the previous TSS. This second transcript defines an intron containing another transcribed locus in the opposite strand, the gene *MAEL*. B) *CNGA3*: Cap-finder RACE of human retina mRNA confirmed the presence of an untranslated exon localised 23,4 kb upstream of exon1. One isoform contains alternatively spliced variant of exon 0 (asterisk). C) *RDH12*: the schematic representation of EST from human retina shows two forms of the transcripts starting in a new retina TSS. These two isoforms contain two alternatively spliced variants of the second exon (asterisk).

- *C1orf32*. This transcribed locus in chromosome 1 was selected for its retinal expression. For this gene, whose function is still not characterised, we retrieved two new isoforms lacking the first annotated exon found in RefSeq. These isoforms contain TSSs in two new exons. One form displays a new first exon located 3 kb downstream from the previous TSS. The other form presents a first exon located 58,4 kb upstream of the former TSS, generating a new first intron spanning a locus transcribed in the opposite strand, the gene *MAEL*. (Figure 1A)

- *CNGA3 *(cyclic nucleotide gated channel alpha 3) codes for the α-subunits of the cone photoreceptor cGMP-gated channel, a crucial component of the cone phototransduction cascade in colour vision. Mutation in this gene causes achromatopsia. The RACE experiment confirmed the presence of 4 isoforms, all containing a splicing of untranslated exon 0 localised 23,4 kb upstream of exon1 [18] (Figure 1B).

- *RDH12 *(Retinol dehydrogenase 12) is an enzyme with dual-specificity retinol dehydrogenases that metabolise both all-*trans*- and *cis-*retinols, reported to be expressed in photoreceptors [19]. Mutations within *RDH12 *cause both recessive early onset Retinitis pigmentosa and Leber's congenital amaurosis [20,21]. In human retinal mRNA we retrieved two forms of the transcripts containing a new first exon located upstream of the RefSeq TSS and a differentially spliced second exon. The *in silico *assembly and experimental pipeline allowed us report three putative TSSs for this gene; the first is defined by the RefSeq annotation, the second was deduced from the most upstream transcript represented by ESTs from pooled colon and the third is a new TSS displayed by retinal transcripts (Figure 1C).

- *DHRS3 *(dehydrogenase/reductase, SDR family, member 3) codes for an enzyme catalysing the reduction of all-*trans*-retinal to all-*trans*-retinol in the presence of NADPH [19,22]. The gene was included in our study for its high expression in retina. Cap-finder RACE confirmed the previous first exon and TSS. We also detected an alternative TSS in a new first exon downstream from the annotated one which was predicted with FirstEF [6]. (Additional file 2: Figure 5).

- *ELOVL5 *(elongation of long chain fatty acids, including docosahexanoic acid (DHA), family member 5). This gene was recently annotated as a retinal expressed gene [23] and a target of mutation studies in retinitis pigmentosa [24]. We detected a new form of the transcript with a new first exon that was not previously annotated or described for retina. (Additional file 3: Figure 6)

- *KIFC3 *(Kinesin family member C3) codes for a retina specific microtubule-associated force-producing protein that may play a role in intracellular transport [25]. We have characterised two new isoforms of *KIFC3 *retinal transcripts which lack the first 3 exons annotated in RefSeq. Both transcripts include a new first exon that localizes these new TSSs 44 kb upstream and 27 kb downstream respectively from the TSS referenced in the RefSeq database. The more upstream start site locates the gene in proximity to another retina specific gene (*CNGB1*). (Additional file 4: Figure 7)

- *RCV1 *(recoverin) inhibits rhodopsin kinase activity in retinal photoreceptors by reducing the binding of arrestin to rhodopsin. Deregulation of recoverin expression in certain types of cancer demonstrates a pathological role in cancer-associated retinopathy [26]. Although a previous study of the promoter was performed [27], no clear evidence of the TSS have been described. For this gene we detected three alternative transcripts; the first with the same 5' end as the previously annotated TSS (first exon length may vary from 203 bp longer to 444 bp shorter), the second with a more frequent isoform lacking the first exon and starting 80 bp upstream from the second exon of the RefSeq and the third form has a new first exon located downstream from the annotated one. (Additional file 5: Figure 8).

- *SLC24A2 *(solute carrier family 24, sodium/potassium/calcium exchanger, member 2) codes for a potassium-dependent sodium-calcium exchanger in cone photoreceptor [28]. Although variant alleles of the cone *SLC24A2 *gene have been identified, none of them are definitively associated with a specific retinal disease [29]. The new model we present for *SLC24A2 *predicts three putative TSSs located in two new additional exons that are alternatively spliced (Additional file 6: Figure 9).

We also investigated whether the new exons that extend the 5' end of the transcript may introduce new potentially protein coding sequences. We didn't observe in any case an extension of the open reading frame beyond the annotated start codon. However short alternate open reading frames of at least 40 codons were observed for *C1orf32 *(nucleotide position 18–290 from the TSS in isoform a, and position 164–400 in isoform b), *CNGA3 *(position 166–315), *DHRS3 *(position 166–315), *KIFC3 *(position 4–195), and *SLC24A2 *(position 55–183 isoform a, 44–289 isoform b). Yet the translated sequences of these short ORFs do not have homology with any protein in public databases.

### Detection of novel splicing variants and shorter transcripts

Our experimental procedure described alternatively spliced isoforms for two genes *IMPG1, SAG*, which lack exon 2 of the RefSeq. These forms have not been annotated in the RefSeq database. We confirmed these alternatively spliced isoforms by regular RT-PCR (Data not shown). The second exon of the gene *SAG *contains the TSS and the presence of this alternative form, lacking the regular start site, may play a role in the regulation and further processing of the transcript. For 4 genes (*CRB2, CRX, RP1, WDR17*), we detected shorter transcripts that lack the annotated start codon. Since these experiments were done with the same adapter ligated first-strand cDNA we assume that these short transcripts are derived from true alternative TSSs. These transcripts may be preferentially amplified in the RT-PCR and may be translated from an internal initiation codon. We report in Table 1 the detailed results for these genes.

### Confirmation of results with primer extension

**Figure 2:**
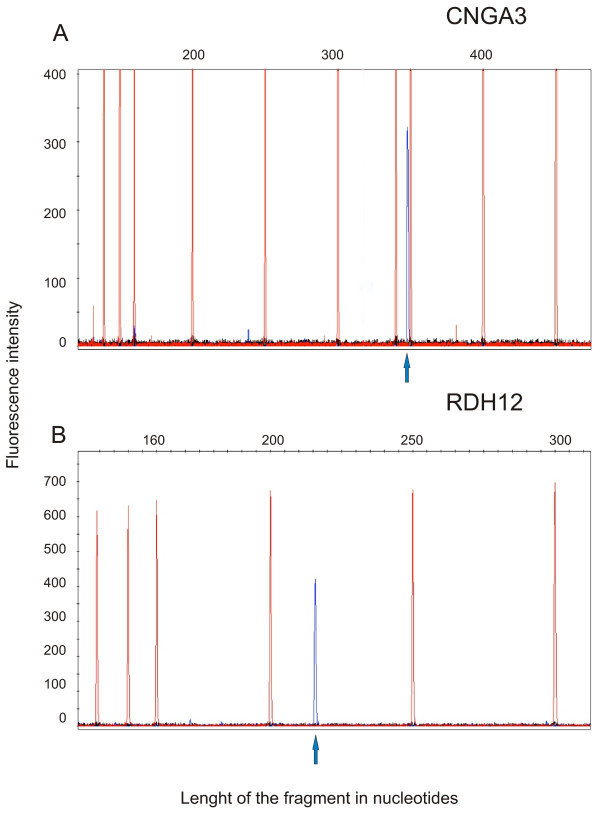
**Primer extension results from WERI-RB1 retina cell lines mRNA**. Primer extension products obtained with the gene specific primer for CNGA3 (A) and for RDH12 (B). The blue peaks in each panel correspond to the primer extension product (FAM-labelled cDNA). The elongation products size 350 bp and 215 bp were respectively expected from the data of RACE cDNA sequences (Blue arrow). Red peaks are the GeneScanR-500 ROX internal lane standards. In the y-axis is indicated the intensity of fluorescence, in the x-axis the number of nucleotides.

To provide an experimental validation of our results we undertook primer extension experiments. We performed reverse transcription of mRNA with a sequence-specific FAM-labelled primer for two genes (*CNGA3, RDH12*). The length of the FAM-labelled cDNA primer extension product can be analysed on ABI-DNA Genetic Analyser using GeneScan software. As a result of the analyses we detected a fragment of 350 bp for *CNGA3 *(Fig 2A) and a fragment of 215 bp for *RDH12 *(Figure 2B). The size of these fragments confirms the presence of the transcripts that we detected with RACE.

### Comparison with existing annotations and databases

To assess the quality of current annotations of the 5' end of genes expressed in human retina, the sequences obtained by 5' RACE were compared with the corresponding gene annotation/prediction. Overall, RACE experiments detected 15 exons that were neither annotated nor predicted for retina transcripts; 8 exons did not have any matching experimental evidence in GenBank, while the other 7 showed different boundaries or alternative splice sites. Of these 15 un-annotated exons, 12 are first exons and can be considered the new first exon for the retinal transcripts. Of the 54 genes successfully amplified, 41 (76%) delivered 5' RACE sequences different from the annotation. Results of a parallel project, DBTSS [8], supported our results concerning 3 of these genes (*CNGA3, ELOVL5 *and *SLC24A2*) although the source of mRNA was not human retina. We extended the annotated first exon of 27 RefSeq genes by an average of 60 transcribed bases. We compared our results with genome wide mapping of TSSs using CAGE tags [10]. We found perfect correspondence for 13 transcript isoforms; for another 6 transcripts the start site retrieved in the CAGE database is located less than 400 bp away. For 35 transcript isoforms the TSS is located in a different position (See Table 1). This discrepancy in the results may be due to the fact that the CAGE database doesn't include retina amongst the panel of analysed tissues and therefore lack specific and rare transcript isoforms present in that tissue.

### Shape of TSSs and conservation

**Figure 3:**
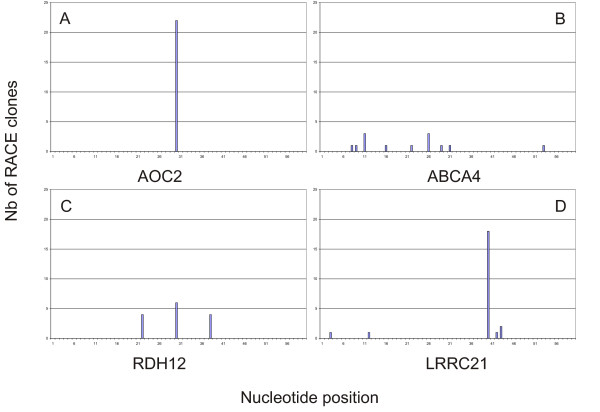
**TSSs present different shapes**. Histograms indicate the number of RACE clones mapping at each nucleotide position. Examples show the different pattern that we observed during the analysis of the Cap-finder RACE. A) Clones distribution for *AOC2 *(single peak class: SP). B) Clones distribution for *ABCA4 *(broad: BR). C) Clones distribution for *RDH12 *(multimodal: MU). D) Clones distribution for *LRRC21 *(broad with dominant peak shape: PB).

After analyzing the distribution of RACE clones we could define the shape of TSSs according to the classification previously reported [10]. The different clones were clustered and depending on the start base position of each clone within a cluster we divided the start sites into four shapes. In the single dominant peak class (SP) the majority of clones are concentrated to no more than four consecutive start positions with a single dominant TSS. The clusters spanning a broader region are grouped in a general broad distribution (BR), a broad distribution with a dominant peak (PB) and a bi- or multimodal distribution (MU): 22 genes showed a single dominant peak, 11 a broad distribution, 8 a bi-multi peak distribution and 6 a broad distribution with a dominant peak. For some transcripts we could not make a classification because the number of clones was less than 5. We report the results of this analysis Table 1 (TSS shape). Figure 3 shows a graphical view of TSSs identified for *AOC2*, *ABCA4*, *RDH12*, and *LRRC21 *as an example of the different distributions observed. The classification of the shape of TSSs defined by distribution of 5' end RACE clones within a cluster is useful for the further characterization of expression regulation. The distribution of the clones defines different elements of the core promoter and gives insights on the start of transcription. Even if broad promoters are the major class in mammals [10], 36 of the analysed transcripts present a dominant peak highlighting the possibility that those transcripts are tightly regulated.

Although TSSs of orthologous genes do not necessary reside on equivalent locations because of evolution of mammalian TSSs [30], we analysed sequence conservation of the first new exons among a set of mammals (mouse, dog, cow): the range of conservation varies between 42 and 89 %. We report the pairwise alignment percentage of identity in Additional file 7.

Sequences residing upstream and downstream from the boundaries of new defined exons are regions displaying high regulatory potential calculated by a computational algorithm [31] integrated in the UCSC genome browser. The regulatory potential (RP) scores computed for the 500 bp sequence upstream the TSS shows that in 9 new first exons out of 11 the RP value exceeds an arbitrary threshold of 0.2 (data not shown). Considering 500 bp downstream the splice site of the first exon the RP value is > 0,2 at least for 7 first exons out of 11. This observation confirms the importance of the new described exons to locate new regulatory elements that are important for transcription in retina.

**Figure 4:**
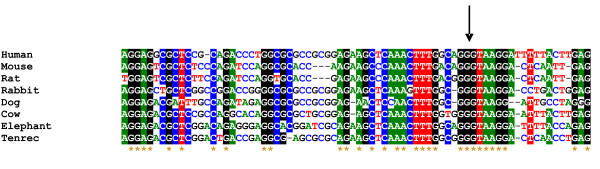
**Inter-species alignment of exon-intron boundaries of the exon 0 of CNGA3**. Conserved nucleotides are labelled with colours and with the star in the bottom those conserved in all the analysed species (human, mouse, rat, rabbit, dog cow, elephant and tenrec). Arrow highlights the splice donor site.

A high level conservation was observed for splice donor sites of the new first exon. 5 genes show an average conservation of at least 75% in the region -3/+5 spanning the splice donor site. For example, we report an *inter*-species alignment of the 3' end of exon 0 (*CNGA3*). The sequence conservation at the level of the splice donor site highlights the possibility of a particular role for this splicing (Figure 4).

## Discussion

Now that the information pertaining to the genomes of human and other animals is available, the next challenge for genetic studies is to map the TSSs and regulatory sequences of the genes. Here, we carried out a study to determine the genuine TSSs of a pool of retinal expressed genes. The results give correct information about the complete 5' end of transcripts and this data will be useful to locate the respective core and proximal promoter elements. We chose Cap-finder RACE to map the TSS of retinal expressed genes because this technique is selective for the complete transcript [8,32]. For 41 out of 54 successfully amplified genes, the Cap-finder RACE experiments detected transcripts which are different from the current gene annotation In most cases the RefSeq was incomplete. Transcripts were missing part of the first exon or even complete exons at the 5' end. This experimental determination of TSSs shows that the current gene annotation was in most cases obtained from data sources that are not strictly selective for the complete transcribed form, and need to be updated. This procedure led us to discover several transcript isoforms that were un-annotated and to locate retina specific TSSs. Proteins encoded by these genes are essential for retina function and stability. A mutation in the *cis*-regulatory elements may influence the level of transcription and have a strong effect due to sensitivity of photoreceptors for high level transcription of genes involved in phototransduction. The new described *cis*-regulatory regions and untranslated exons are possible targets for mutation studies in retinal disorders. Therefore new isoforms give a more complete picture of alternative start sites use in retina genes. The 5' untranslated region may contain important transcriptional and post transcriptional regulatory sites [33-35] and therefore only the complete 5' UTR provides the opportunity to study the potential regulatory role of these non-coding sequences. New reported TSSs contribute to the identification of regulatory elements active in tissue specific gene regulation [36,37]. Moreover, bioinformatics tools that identify common regulatory elements rely on the correct determination of TSSs within a particular tissue [38], therefore these computational approaches will only be effective after experimental validation of the 5' end of transcripts.

## Conclusion

We herein report the TSSs for 54 retina expressed genes. Our results define new and more precise locations of TSSs for 76% of the analysed genes; moreover in 15% of the genes we found new exons in the 5' end of the transcripts. Thus, this analysis of TSSs in human retina was essential to define the complex pattern of transcripts present in this tissue.

Our results highlight the importance of applying a tissue specific approach with a systematic program of Cap-finder RACE using the known gene structures as a starting point and/or gene predictions to complete the existing gene annotation. The new TSSs and transcribed sequences provide crucial information for further exploration of the promoter and other *cis-*regulatory sequences at the 5'end of the gene, and in particular for the study those elements that are functionally active in human retina.

## Methods

### *In silico *analysis

We used available public database information (RefSeq, NCBI and Ensemble predictions covered by at least one EST, Unigene EST database) to perform *in silico *assembly and analysis of 5'transcript termini. The procedure involves downloading the sequences from public databases, clustering them to obtain a consensus and designing a gene model with the most complete 5' transcript termini. Sequences were downloaded and assembled using the SeqMan program (DNAstar). In our analysis we consider retina ESTs as well as non-retinal ESTs to obtain the most complete information about the different alternative start sites already mapped. Additionally, the information about putative TSS was assessed using Promoser [39], Eukaryotic Promoter Database Current Release 87 [40], and Database of Transcriptional Start Sites DBTSS [41]. First exon boundaries were determined by aligning the predicted sequence to the genome using BLAT [42]. The gene model was then deduced considering the clustering of all the collected sequences giving the priority to the most accurate database in the 5' termini (EPD and DBTSS), but considering also gene prediction (GENSCAN, implemented in the UCSC Genome Browser) and gene annotation that are confirm by at least one EST [43]. To evaluate first exons conservation we used BioEdit pairwise and multiple alignments [44]

### Primer design

Gene specific reverse primers were selected within exons other than the first exon to obtain spliced products. Two primers were chosen for each gene, in order to perform a nested PCR, which allows to enhance specificity, and to obtain a sufficient amplification product for rare transcripts. Primers were designed using the Primer3 software [45], and checked for uniqueness by querying against the human genome.

### RACE protocol

We applied the RNA-ligase-mediated RACE (RLM-RACE) system from Ambion. RNA sample from adult human retina was treated with DNAse I. After DNase treatment and inactivation, 10 μg of total RNA was dephosphorylated for 60 min at 37°C with 10 U Calf Intestinal Phosphatase to remove the 5'-phosphate from all RNA species except those that have a cap structure (present on all Pol II transcripts). RNA was then phenol/chloroform extracted, precipitated and re-suspended in water. Dephosphorylated RNA was then digested for 60 min at 37°C in a 10-μL reaction with 10 U tobacco acid pyrophosphatase. Subsequently the RNA was incubated for 60 min at 37°C with 1 U T4 RNA ligase and 0.3 μg of an RNA adapter (5' RACE Adapter 5'-GCU-GAU-GGC-GAU-GAA-UGA-ACA-CUG-CGU-UUG-CUG-GCU-UUG-AUG-AAA-3'). After ligation, 180 ng of RNA was incubated for 2 min at 75°C in the presence of 5 μM random decamers in RT buffer. Single-stranded cDNA was generated by the addition of 100 U M-MLV RT and incubation at 42°C for 60 min.

Amplification of 5' RACE cDNA was carried out using nested gene-specific primers and adapter specific primers and with 1 μl of the first-strand cDNA reaction. PCR reactions were done in 50 μl volume including 5 μl 10× PCR Buffer supplied in the RLM kit, 4 μl dNTP Mix 10 mM, 2 μl 5' RACE gene-specific outer primer (10 μM), 2 μl 5' RACE Outer or Inner Primer (10 μM), and 1 U thermostable DNA polymerase. Cycling conditions were: 5 min initial denaturation at 94°C PCR followed by 35 cycles of 95°C for 30 s, 60-55°C (empirically determined) for 30 s (annealing), 72°C for 30 s (extension) and a final extension at 72°C for 7 mins. Amplified products were analysed on a 3% agarose gel and visualised by ethidium bromide staining.

### Cloning and sequencing of RACE products

5' RACE products were cloned into pCR-2.1 vector (Invitrogen). 2 μL of PCR reaction were incubated with 0.1 ng of vector at 16°C over-night. Aliquots of the ligation were used to transform library efficiency chemically competent E. coli DH5α (Invitrogen). 8 to 48 clones of each transformation were subjected to colony PCR and the inserts sequenced with standard M13 forward and reverse primers applying Big Dye Terminator v3.0 chemistry (Applied Biosystems). Sequencing products were separated and analysed on an ABI 3100 DNA sequencer.

### Sequence analysis

Gene sequences and cDNA sequences, obtained using the RACE, were aligned to the human genome using BLAT. cDNA sequence was considered informative only if the following criteria were met: (I) the spliced sequence mapped to the same region of the genome as the gene sequence; (II) the product could be mapped uniquely to the genome with >95% identity and (III) presence of the gene specific primer sequence and the 5' RACE Adapter primer sequence.

### Primer extension

A fluorescein phosphoramidites (FAM)-labelled reverse primer was added to 10 μg of DNAseI-treated total RNA to a final concentration of 5 nM. Samples were heated at 70°C for 5 min followed by 20 min incubation at 58°C and then allowed to cool for 15 min to room temperature. First strand cDNA synthesis was performed using Avian Myeloblastoma Virus (AMV) reverse transcriptase and 5× AMV-RT buffer (Promega) according to the manufacturer's instructions in a final volume of 60 μl. The primer extension reaction was done in two repeated reaction cycles. After an initial reverse transcription step (60 min at 42°C in a total volume of 50 μl), enzyme was replenished and the samples underwent a second extension reaction (60 min at 42°C, adjusting the buffer to a total volume of 60 μl). Finally 3 μl of 5 M NaOH was added to each cDNA sample, the reaction incubated at 37°C for 15 min and then neutralised with 16 μl of 2 M HEPES free acid. Extension products were purified using the columns AutoSeq G-50 (Amersham Pharmacia Biotech). Samples were separated on 50-cm capillary columns in the POP4 acryl amide polymer (Applied Biosystems) on an ABI PRISM 3100 Genetic Analyser (Applied-Biosystems) Sequencer with GENESCAN 500-ROX added as size standard (Applied-Biosystems). The sequences of the gene specific reverse primers were: *RDH12 *5' tgagcagcagcctgactctgagcaga gcccaga 3', *CNGA3 *5'atcttctcggtttgtcacatttagc 3', with 5' ends modified with the fluorescent molecule 6-FAM.

## Authors' contributions

VR contributed to acquisition, analysis, interpretation of data and drafting the manuscript; RC has been involved in acquisition, analysis and interpretation of data and drafting the manuscript; BW conceived and coordinated the study and contributed materials and resources. All authors read and approved the final manuscript.

## Supplementary Material

Additional File 1 **List of genes selected for the study of identification of TSS**. We provide in the list the Gene Symbol, Gene Name, Chromosomal location, Tissue/cell type of expression, associated disease of the genes selected for our study. Abbreviation used in the table: RP: retinitis pigmentosa, CRD: cone-rod dystrophy, FF: fundus flavimaculatus, MD: macula degeneration, RD: retinal dystrophy. Click here for file

Additional File 2 **Figure 5: Schematic gene structure of *DHRS3***. Schema of the RefSeq, ESTs, exonic structure of new isoforms identified with Cap-finder RACE of human retina mRNA and a schema of the new genomic structure containing new TSSs (indicated by red arrows). The Cap-finder RACE allow us to confirm the first exon and TSS of this gene. We also show the presence in retina of an alternative form of transcript containing a first exon 21 kb downstream the annotated TSS. Click here for file

Additional File 3 **Figure 6: Schematic gene structure of *ELOVL5***. Schema of the RefSeq, ESTs, exonic structure of new isoforms identified with Cap-finder RACE of human retina mRNA and a schema of the new genomic structure containing new TSSs (indicated by red arrows): we show the presence in retina of an alternative transcript with a first exon downstream from the annotated one. Click here for file

Additional File 4 **Figure 7: Schematic gene structure of *KIFC3***. Schema of the RefSeq, ESTs, exonic structure of new isoforms identified with Cap-finder RACE of human retina mRNA and a schema of the new genomic structure containing the TSSs (indicated by red arrows): figure shows two new isoforms lacking the 3 first exons annotated in the RefSeq. Both transcripts let us to define new TSSs located respectively 44 kb upstream and 27 kb downstream from the previous TSS. Click here for file

Additional File 5 **Figure 8: Schematic gene structure of *RCV1***. Schema of the RefSeq, ESTs, exonic structure of new isoforms identified with Cap-finder RACE of human retina mRNA and a schema of the new genomic structure containing new TSSs (indicated by red arrows). The figure shows three isoforms that we obtained with RACE experiments: one form extending the first exon by 203 bp and the other two forms lacking the first annotated exons of the RefSeq and, respectively, containing one new first exon that splices with the second annotated one and one starting at the second exon but extending it by 80 bp. For both transcripts the TSS is downstream from the annotated one. Click here for file

Additional File 6 **Figure 9: Schematic gene structure of *SLC24A2***. Schema of the RefSeq, ESTs, exonic structure of new isoforms identified with Cap-finder RACE of human retina mRNA and a schema of the new genomic structure containing new TSSs (indicated by red arrows): 4 new cDNAs clones from human retina identify 4 new TSSs for this gene. The transcripts contain two additional exons, a and b, that are alternatively spliced. Click here for file

Additional File 7 **Sequence conservation of new first exon**. Analysis of sequence conservation of the first new exons of the listed human transcript in comparison with a set of mammals (mouse, dog, cow). Numbers indicate percentage of identity. Click here for file

## Figures and Tables

**Table 1:** Results of the RACE experiment to determine the TSS of retina transcripts

**Gene Symbol**	**RACE results**		**TSS location**			**Chr.**	**Shape**	**CAGE**
						
			**Start**	**Int. peak**	**End**			
***C1orf32***	Two new first exons	Isoform a	165,208,791			1q24.1	**SP**	**+**
		Isoform b	165,269,346				**-**	**-**

	Extension exon 0 of 180 bp	Isoform a	98,329,050		98,329,083	2q11.2	**BR**	**+**
***CNGA3***		Isoform b	98,329,288				**SP**	**-**
		Isoform c	98,329,371				**SP**	**+**

***DHRS3***	New first exon	Isoform a	12,578,722			1p36.1	**SP**	**-**
	Shorter exon 1 of 508 bp	Isoform b	12,599,903		12,599,935		**SP**	**-**

***ELOVL5***	New first Exon		53,320,946			6p21.1-p12.1	**-**	**-**

***KIFC3***	Two new first exons	Isoform a	56,437,970			16q13-q21	**SP**	**-**
		Isoform b	56,370,953				**SP**	**-**

***RCV1***	Extension exon 1 of 203 bp	Isoform a	9,749,613	9,749,402	9,748,934	17p13.1	**PB**	**-**
	New first Exon	Isoform b	9,745,910				**-**	**-**
	Lack of exon 1. exon 2 extended	Isoform c	9,745,244		9,745,221		**MU**	**-**

***RDH12***	Two new exons		67,254,268	67,254,276	67,257,284	14q24.1	**MU**	**-**

		Isoform c	19,778,103			9p22-p13	**SP**	**-**
***SLC24A2***	Two new first exons	Isoform a	19,778,808				**-**	**-**
		Isoform b	19,778,609				**-**	**+**
		Isoform d	19,776,949		19,777,002		**PB**	**+**

	Lack of exon 2		76,839,115		76,839,078	6q14.2-q15	**SP**	**-**
***IMPG1***	Lack of exon 1. shortening exon2		76,808,496				**SP**	**-**
	Complete in the first 3 Exons		76,839,060				**-**	**-**

***SAG***	Lack of exon 2		233,998,462		233,998,525	2q37.1	**BR**	**-**

***AIPL1***		61 bp	6,279,208		6,279,306	17p13.1	**-**	**-**
***AOC2***		10 bp	38,250,126			17q21	**SP**	**+**
***CLUL1***		31 bp	606,669	606,693	606,698	18p11.32	**PB**	**-**
***CNGB3***		43 bp	88,404,286		88,404,354	8q21-q22	**-**	**-**
***EYA3***		14 bp	28,287,732		28,287,684	1p36	**BR**	**+**
***FSCN2***		66 bp	77,109,947		77,109,997	17q25	**-**	**-**
***GNAT1***		44 bp	50,204,027		50,204,047	3p21	**-**	**-**
***GRK7***		40 bp	142,979,632		142,979,726	3q21-q23	**BR**	**-**
***GUCA1B***		19 bp	42,270,691	42,270,689-42,270,672	42,270,649	6p21.1	**PB**	**-**
***GUCY2D***		30 bp	7,846,687			17p13.1	**-**	**-**
***HPCA***		15 bp	33,124,670		33,124,680	2p25.1	**MU**	**+**
***IMPDH1***		206 bp	127,837,736			7q31.3-q32	**SP**	**+**
***IMPG2***		23 bp	102,522,132		102,522,102	3q12.2-q12.3	**BR**	**-**
***LRRC21***	Extension exon 1 of	121 bp	85,991,318	85,991,280	85,991,194	10q23	**PB**	**-**
***MPP4***		30 bp	202,271,692	202,271,616	202,271,601	2q33.2	**PB**	**-**
***OPN4***		67 bp	88,404,287			10q22	**SP**	**-**
***PDC***		17 bp	184,696,879		184,696,869	1q25.2	**MU**	**-**
***PDE6H***		7 bp	15,017,238		15,017,243	12p13	**SP**	**-**
***PRPF31***		117 bp	59,310,532		59,310,609	19q13.42	**MU**	**-**
***RdCVF***		37 bp	17,432,763	17,432,753-17,432,735	17,432,716	19p13.11	**MU**	**-**
***RDH5***		39 bp	54,400,449			12q13-q14	**SP**	**-**
***RDH8***		63 bp	9,984,862	9,984,925	9,984,991	19p13.2-p13.3	**MU**	**-**
***RDS***		61 bp	42,798,348		42,798,296	6p21.2-p12.3	**BR**	**-**
***RLBP1***		83 bp	87,566,008		87,565888	15q26	**-**	**-**
***ROM1***		215 bp	62,136,883		62,137,111	11q13	**BR**	**-**
***RPGR***		7 bp	38,071,739		38,071,704	Xp11.4	**BR**	**-**
***TULP1***		70 bp	35,588,693		35,588,651	6p21.3	**PB**	**+**

***CRB2***		52 bp	125,158,322		125,158,324	9q33.2	**-**	**-**
***CRX***	Shortening exon 1 of	60 bp	53,016,971	53,017,001	53,017,005	19q13.3	**SP**	**-**
***RP1***		53 bp	55,691,206		55,691,233	8q11-q13	**BR**	**+**
***WDR17***		77 bp	177,224,132	177,224,169-177,224,185	177,224,202	4q34	**MU**	**+**

***ABCA4***			94,359,290		94,359,245	1p22.1-p21	**BR**	**-**
***C14ORF2***			103,457,619			14q32.33	**-**	**+**
***C1QL2***			119,632,934			2q14.2	**-**	**-**
***C7orf9***			25,427,912			7p21-p15	**-**	**-**
***CHM***			85,189,194		85,189,222	Xq21.2	**BR**	**+**
***ELOVL4***			80,714,034			6q14	**-**	**+**
***OPN1SW***	Previous 5' end confirmed		128,203,084			7q31.3-q32	**SP**	**-**
***RAX***			55,091,570			18q21.32	**SP**	**-**
***RBP3***			48,010,989		48,011,000	10q11.2	**-**	**-**
***RDH11***			67,232,201		67,232,186	14q24.1	**SP**	**-**
***RHO***			130,730,174			3q21	**SP**	**-**
***RPL14***			40,473,831			3p22	**SP**	**+**
***RS1***			18,600,144			Xp22.2-p22.1	**SP**	**-**

The Table provides Gene Symbols of the processed genes, RACE results referring to the RefSeq entry (database release 18), nucleotide position of the TSS (start, internal frequent start and end) referring to the UCSC Human Genome Browser (March 2006 assembly), Chromosomal location (Chr.), shape of the TSS (Shape) according to the classification of Carninci et al., 2006: single peak [SP], broad [BR], bimodal/multimodal [MU], broad with dominant peak shape [PB], comparison with Cage TSS database (+ for correspondence, – no correspondence). The genes are listed according to the type of results that was obtained according to the description in the text: (i) new TSSs within novel exons (8 genes), (ii) alternative splice form of the second exon (2 genes:, (iii) extension of the annotated first exon (27 genes), (iv) length shortening of the annotated first exon (4 genes), (v) confirmation of previously annotated TSSs (13 genes).
