# Supplementary material for: Mapping of transcription start sites of human retina expressed genes
Source: BMC Genomics. 2007 Feb 7;8:42. doi: 10.1186/1471-2164-8-42 (PMC1802077; doi:10.1186/1471-2164-8-42)
Supplement: Additional File 1 — List of genes selected for the study of identification of TSS. We provide in the list the Gene Symbol, Gene Name, Chromosomal location, Tissue/cell type of expression, associated disease of the genes selected for our study. Abbreviation used in the table: RP: retinitis pigmentosa, CRD: cone-rod dystrophy, FF: fundus flavimaculatus, MD: macula degeneration, RD: retinal dystrophy. [file 1471-2164-8-42-S1.doc]

| **List of the genes selected for Cap-finder RACE procedure** | |  |  |  |
| --- | --- | --- | --- | --- |
| **GeneSymbol** | **Gene Name** | **Chromosomal location** | **Tissue of expression** | **Associacion with disease** |
| ***ABCA4*** | ATPbinding cassette, subfamily A (ABC1), member 4 | 1p22.1p21 | Photoreceptors | Stargardt disease, FF, CRD, MD age related, RP |
| ***AIPL1*** | aryl hydrocarbon receptor interacting proteinlike 1 | 17p13.1 | Photoreceptors, pineal gland | recessive Leber congenital amaurosis; dominant CRD |
| ***ANKRD33*** | ankyrin repeat domain 33 | 12q13.13 | Retina | n/a |
| ***AOC2*** | amine oxidase, copper containing 2 (retinaspecific) | 17q21 | Retinal ganglion cell layer | Choroideremia |
| ***C14ORF2*** | chromosome 14 open reading frame 2 | 14q32.33 | Ubiquitous | n/a |
| ***C1orf32*** | chromosome 1 open reading frame 32 | 1q24.1 | Eye | n/a |
| ***C1QL2*** | complement component 1, q subcomponentlike 2 | 2q14.2 | Eye, Brain | n/a |
| ***C7orf9*** | chromosome 7 open reading frame 9 | 7p21p15 | Retina | n/a |
| ***CHM*** | choroideremia (Rab escort protein 1) | Xq21.2 | Ubiquitous | Choroideremia |
| ***CLUL1*** | clusterinlike 1 (retinal) | 18p11.32 | Retina (cones) | n/a |
| ***CNGA3*** | cyclic nucleotide gated channel alpha 3 | 2q11.2 | Retina (cones), pancreas, brain, testis | Achromatopsia 2 |
| ***CNGB3*** | cyclic nucleotide gated channel beta 3 | 8q21q22 | Retina (cones), skeletal muscle, pineal gland, brain | Achromatopsia 3 |
| ***CRB1*** | crumbs homolog 1 (Drosophila) | 1q31q32.1 | Photoreceptors (inner segment) | Leber congenital amaurosis |
| ***CRB2*** | crumbs homolog 2 (Drosophila) | 9q33.2 | Retina | n/a |
| ***CRX*** | conerod homeobox | 19q13.3 | Photoreceptors | CRD 2 |
| ***DHRS3*** | dehydrogenase/reductase (SDR family) member 3 | 1p36.1 | Ubiquitous, high expression in retina | n/a |
| ***ELOVL4*** | elongation of very long chain fatty acids (FEN1/Elo2, SUR4/Elo3, yeast)like 4 | 6q14 | Photoreceptors (inner segment) | MD, autosomal dominant, chromosome 6 linked |
| ***ELOVL5*** | elongation of long chain fatty acids (FEN1/Elo2, SUR4/Elo3like, yeast) like 5 | 6p21.1p12.1 | Ubiquitous | n/a |
| ***EYA3*** | eyes absent homolog 3 (Drosophila) | 1p36 | Ubiquitous | n/a |
| ***FSCN2*** | fascin homolog 2, actinbundling protein, retinal (Strongylocentrotus purpuratus) | 17q25 | Eye, tyroid, placenta,ovary | RP 30 |
| ***GNAT1*** | guanine nucleotide binding protein (G protein), alpha transducing activity polypeptide 1 | 3p21 | Retina (rods) | Night blindness, congenital stationary |
| ***GNG13*** | guanine nucleotide binding protein (G protein), gamma 13 | 16p13.3 | Retina Bipolar Cells | n/a |
| ***GNGT1*** | guanine nucleotide binding protein (G protein), gamma transducing activity polypeptide 1 | 7q21.3 | Retina (rods) | n/a |
| ***GRK7*** | G proteincoupled receptor kinase 7 | 3q21q23 | Retina, brain | n/a |
| ***GUCA1A*** | guanylate cyclase activator 1A (retina) | 6p21.1 | Retina, brain and testis | Cone dystrophy 3 |
| ***GUCA1B*** | guanylate cyclase activator 1B (retina) | 6p21.1 | Retina (photorreceptors) | n/a |
| ***GUCY2D*** | guanylate cyclase 2D, membrane (retinaspecific) | 17p13.1 | Retina | CRD 6 |
| ***GUCY2F*** | guanylate cyclase 2F, retinal | Xq22 | Retina | n/a |
| ***HPCA*** | hippocalcin | 2p25.1 | Ubiquitous | n/a |
| ***Hs.221513*** | Clone c222389 mRNA sequence | 6q21 | Retina, testis | n/a |
| ***IMPDH1*** | IMP (inosine monophosphate) dehydrogenase 1 | 7q31.3q32 | Ubiquitous adult, restricted to liver and eye in fetus | RP 10 |
| ***IMPG1*** | interphotoreceptor matrix proteoglycan1 | 6q14.2-q15 | Interface between photoreceptors and the RPE | n/a |
| ***IMPG2*** | interphotoreceptor matrix proteoglycan2 | 3q12.2q12.3 | Interface between photoreceptors and the RPE | n/a |
| ***KIFC3*** | kinesin family member C3 | 16q13q21 | Ubiquitous | n/a |
| ***LHX3*** | LIM homeobox 3 | 9q34.3 | Retina, brain | Pituitary hormone deficiency with rigid cervical spine |
| ***LRRC21*** | leucine rich repeat containing 21 | 10q23 | Photoreceptors (outer segment), brain | n/a |
| ***MPP4*** | membrane protein, palmitoylated 4 (MAGUK p55 subfamily member 4) | 2q33.2 | Retina | n/a |
| ***NR2E3*** | nuclear receptor subfamily 2, group E, member 3 | 15q22.32 | Photoreceptors | Enhanced Scone syndrome |
| ***NRL*** | neural retina leucine zipper | 14q11.1q11.2 | Retina (rods) | RD, autosomal recessive, clumped pigment type |
| ***OPN1SW*** | opsin 1 (cone pigments), shortwavesensitive (color blindness, tritan) | 7q31.3q32 | Cones | Color-blindness, tritan |
| ***OPN4*** | opsin 4 (melanopsin) | 10q22 | Retina (ganglion cells) | n/a |
| ***OPTC*** | opticin | 1q32.1 | Eye | n/a |
| ***OTX2*** | orthodenticle homolog 2 (Drosophila) | 14q21q22 | Retina, brain | n/a |
| ***PCDH21*** | protocadherin 21 | 10q22.1q22.3 | Sensory neurons | n/a |
| ***PDC*** | phosducin | 1q25.2 | Rods | n/a |
| ***PDE6B*** | phosphodiesterase 6B, cGMPspecific, rod, beta | 4p16.3 | Retina (photoreceptor cells) | Night blindness, congenital stationary, type3 |
| ***PDE6G*** | phosphodiesterase 6G, cGMPspecific, rod, gamma | 17q25 | Ubiquitous, in retina resticted to rods | n/a |
| ***PDE6H*** | phosphodiesterase 6H | 12p13 | Retina (cones) | n/a |
| ***PRPF31*** | premRNA processing factor 31 homolog (yeast) | 19q13.42 | Ubiquitous | RP11 |
| ***RAX*** | retina and anterior neural fold homeobox | 18q21.32 | Eye | n/a |
| ***RBP3*** | retinol binding protein 3, interstitial | 10q11.2 | Interface between photoreceptors and the RPE | n/a |
| ***RCV1*** | recoverin | 17p13.1 | Retina and brain | n/a |
| ***RdCVF*** | thioredoxinlike 6 | 19p13.11 | Rods | n/a |
| ***RDH11*** | retinol dehydrogenase 11 (alltrans and 9cis) | 14q24.1 | Ubiquitous and Muller cells | n/a |
| ***RDH12*** | retinol dehydrogenase 12 (alltrans and 9cis) | 14q24.1 | Retina (photoreceptor cells) | Leber congenital amaurosis, type III |
| ***RDH5*** | retinol dehydrogenase 5 (11cis and 9cis) | 12q13q14 | Retinal Pigment Epithelium | Fundus albipunctatus |
| ***RDH8*** | retinol dehydrogenase 8 (alltrans) | 19p13.2p13.3 | Retina (photoreceptor cells) | n/a |
| ***RDS*** | retinal degeneration, slow | 6p21.2p12.3 | Retina (photoreceptor cells) | Butterfly dystrophy, retinal |
| ***RGR*** | retinal G protein coupled receptor | 10q23 | RPE and Muller cells, muscle and brain | RP, autosomal dominant |
| ***RHO*** | rhodopsin (opsin 2, rod pigment) (retinitis pigmentosa 4, autosomal dominant) | 3q21 | Retina (rods) | Night blindness, congenital stationery |
| ***RLBP1*** | retinaldehyde binding protein 1 | 15q26 | RPE | Bothnia retinal dystrophy |
| ***ROM1*** | retinal outer segment membrane protein 1 | 11q13 | Retina (photoreceptor cells) | RP, digenic |
| ***RP1*** | retinitis pigmentosa 1 (autosomal dominant) | 8q11q13 | Retina, testis, muscle, trachea | RP |
| ***RP1L1*** | retinitis pigmentosa 1like 1 | 8p23 | Retina (photoreceptor cells) | n/a |
| ***RPE65*** | retinal pigment epitheliumspecific protein 65kDa | 1p31 | Retina (cones) and RPE | Leber congenital amaurosis 2 |
| ***RPGR*** | retinitis pigmentosa GTPase regulator | Xp11.4 | Retina (photoreceptor cells) | Cone dystrophy 1 |
| ***RPGRIP1*** | retinitis pigmentosa GTPase regulator interacting protein 1 | 14q11 | Ubiquitous, in retina resticted to photoreceptors | CRD 9 |
| ***RPL14*** | ribosomal protein L14 | 3p22 | Ubiquitous | n/a |
| ***RS1*** | retinoschisis (X linked, juvenile) 1 | Xp22.2p22.1 | Secreted photoreceptor protein | Retinoschisis |
| ***SAG*** | S antigen; retina and pineal gland (arrestin) | 2q37.1 | Retina, pineal gland | Oguchi disease1 |
| ***SLC1A7*** | solute carrier family 1 (glutamate transporter), member 7 | 1p32.3 | Ubiquitous high expression in retina | n/a |
| ***SLC24A1*** | solute carrier family 24 (sodium/potassium/calcium exchanger), member 1 | 15q22 | Retina (rods) | n/a |
| ***SLC24A2*** | solute carrier family 24 (sodium/potassium/calcium exchanger), member 2 | 9p22p13 | Retina (cones) | n/a |
| ***TULP1*** | tubby like protein 1 | 6p21.3 | Retina, testis, muscle, larynx | RP 14 |
| ***VAX2*** | ventral anterior homeobox 2 | 2p13 | Ventral portion of the retina (development) | n/a |
| ***WDR17*** | WD repeat domain 17 | 4q34 | Retina, testis | n/a |
