# Supplementary figures and images for: Mapping of transcription start sites of human retina expressed genes
Source: BMC Genomics. 2007 Feb 7;8:42. doi: 10.1186/1471-2164-8-42 (PMC1802077; doi:10.1186/1471-2164-8-42)

# DHRS3

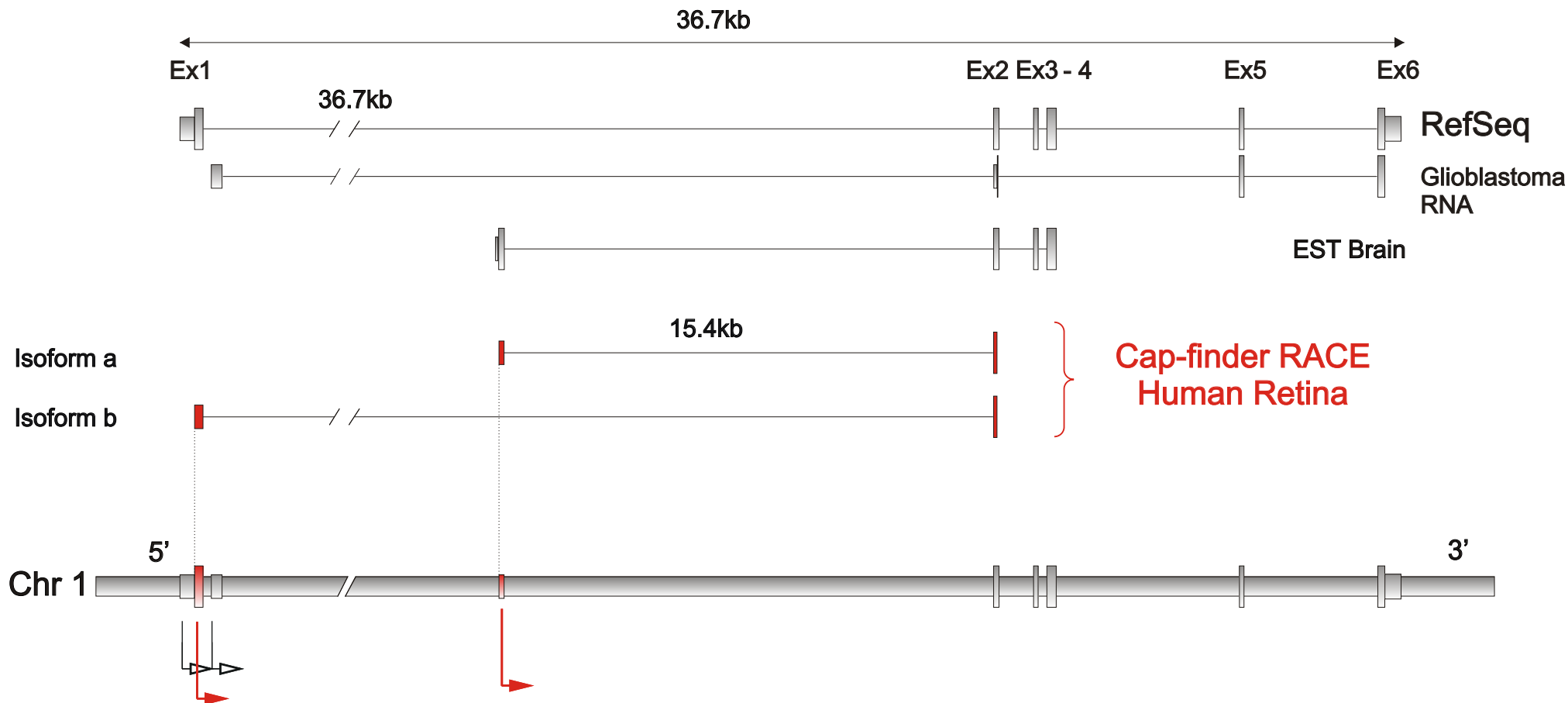

Supplement: Additional File 2 — Figure 5: Schematic gene structure of DHRS3. Schema of the RefSeq, ESTs, exonic structure of new isoforms identified with Cap-finder RACE of human retina mRNA and a schema of the new genomic structure containing new TSSs (indicated by red arrows). The Cap-finder RACE allow us to confirm the first exon and TSS of this gene. We also show the presence in retina of an alternative form of transcript containing a first exon 21 kb downstream the annotated TSS. [file 1471-2164-8-42-S2.pdf]

# *ELOVL5*

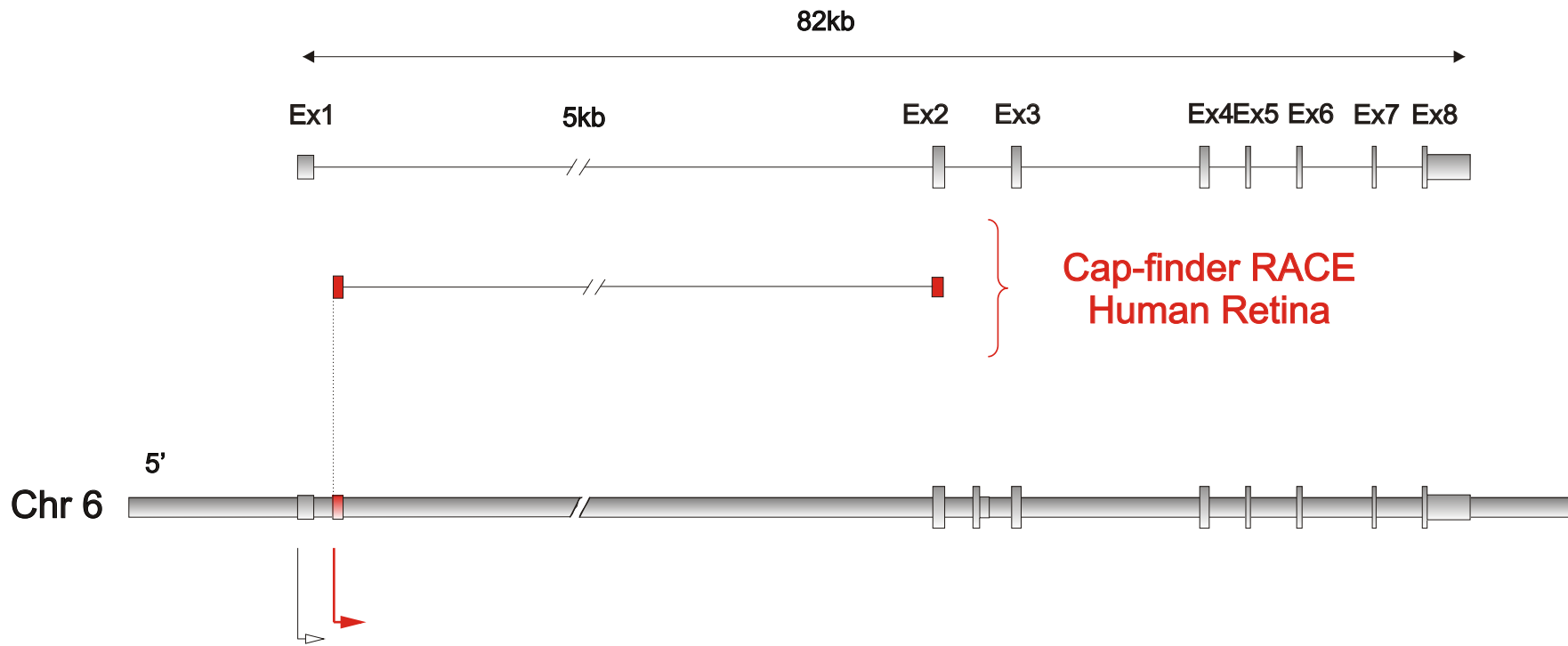

Supplement: Additional File 3 — Figure 6: Schematic gene structure of ELOVL5. Schema of the RefSeq, ESTs, exonic structure of new isoforms identified with Cap-finder RACE of human retina mRNA and a schema of the new genomic structure containing new TSSs (indicated by red arrows): we show the presence in retina of an alternative transcript with a first exon downstream from the annotated one. [file 1471-2164-8-42-S3.pdf]

# KIFC3

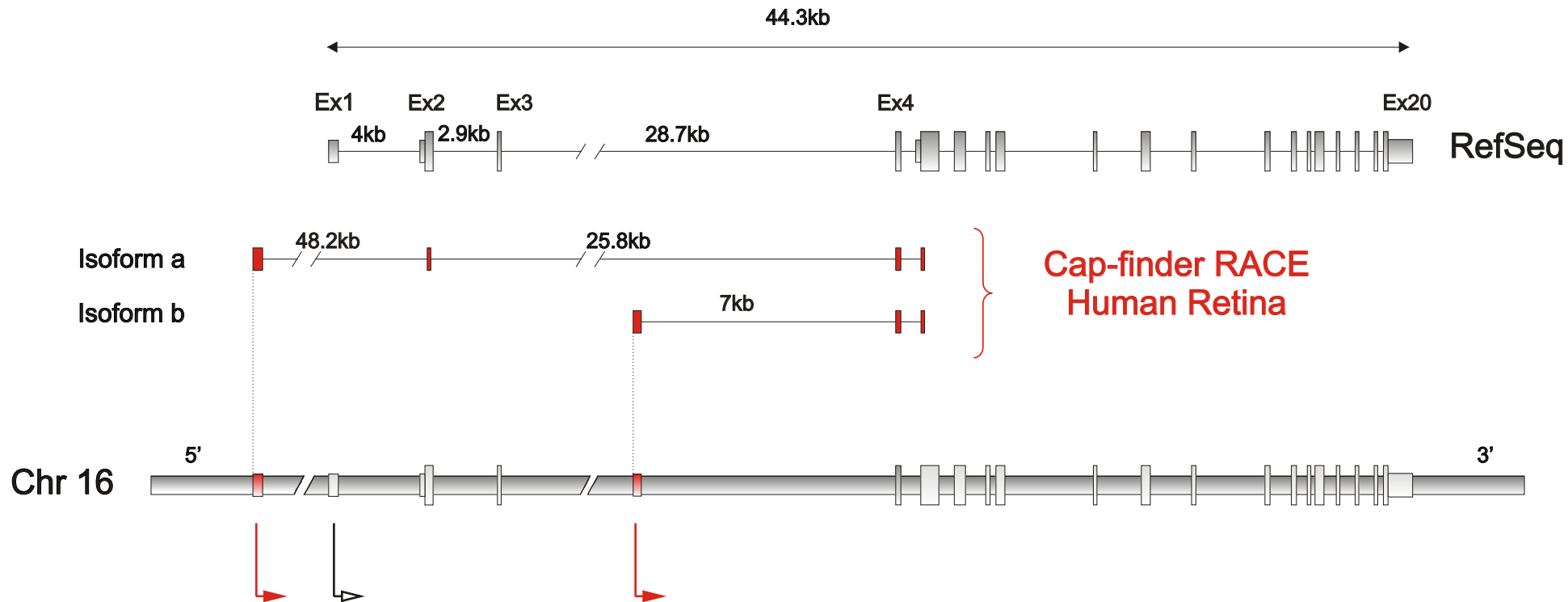

Supplement: Additional File 4 — Figure 7: Schematic gene structure of KIFC3. Schema of the RefSeq, ESTs, exonic structure of new isoforms identified with Cap-finder RACE of human retina mRNA and a schema of the new genomic structure containing the TSSs (indicated by red arrows): figure shows two new isoforms lacking the 3 first exons annotated in the RefSeq. Both transcripts let us to define new TSSs located respectively 44 kb upstream and 27 kb downstream from the previous TSS. [file 1471-2164-8-42-S4.pdf]

# RCV1

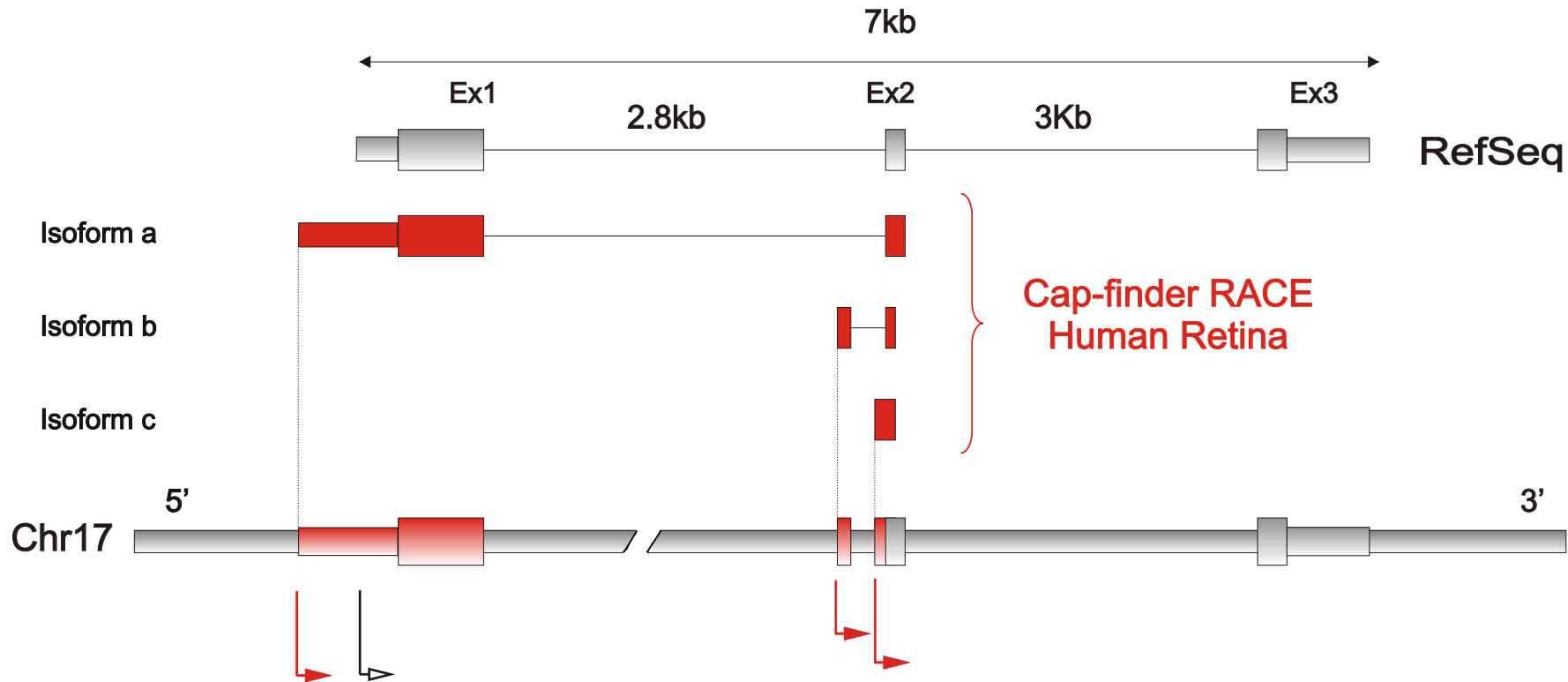

Supplement: Additional File 5 — Figure 8: Schematic gene structure of RCV1. Schema of the RefSeq, ESTs, exonic structure of new isoforms identified with Cap-finder RACE of human retina mRNA and a schema of the new genomic structure containing new TSSs (indicated by red arrows). The figure shows three isoforms that we obtained with RACE experiments: one form extending the first exon by 203 bp and the other two forms lacking the first annotated exons of the RefSeq and, respectively, containing one new first exon that splices with the second annotated one and one starting at the second exon but extending it by 80 bp. For both transcripts the TSS is downstream from the annotated one. [file 1471-2164-8-42-S5.pdf]

# SLC24A2

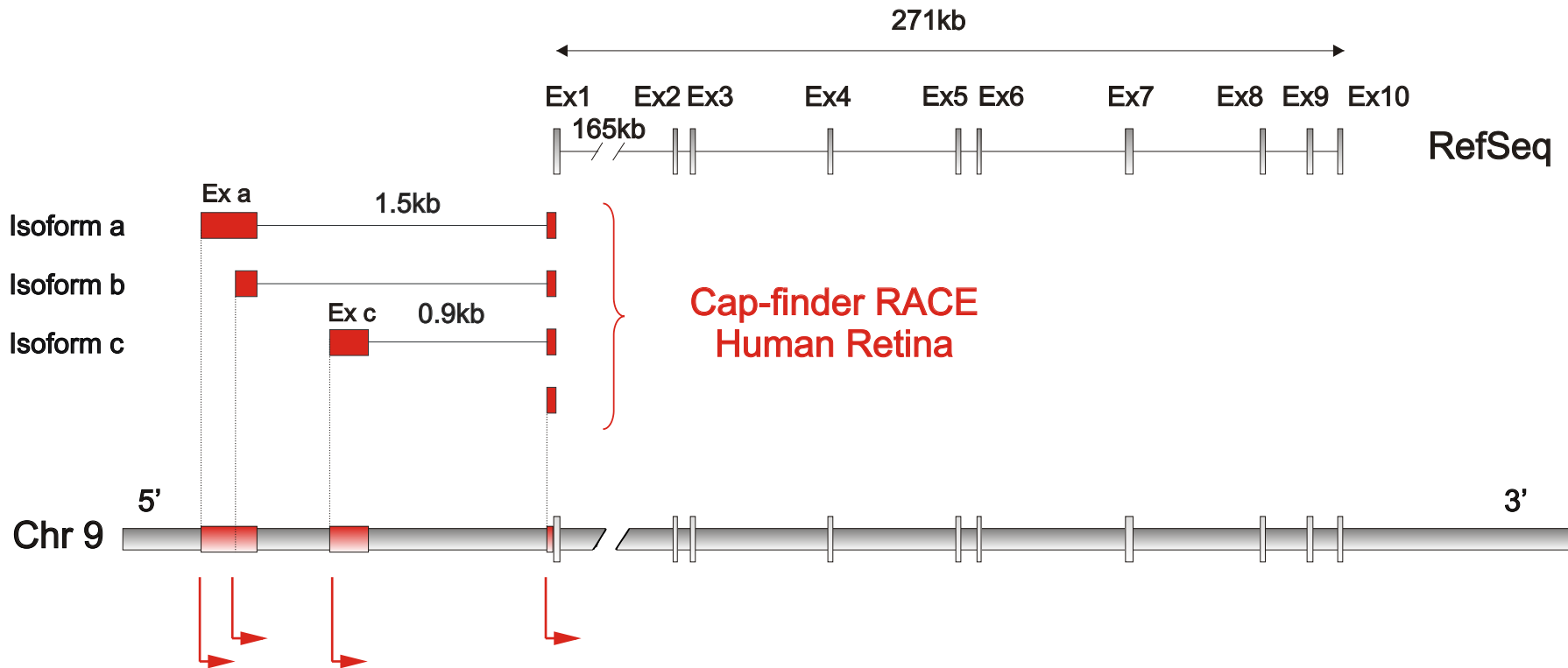

Supplement: Additional File 6 — Figure 9: Schematic gene structure of SLC24A2. Schema of the RefSeq, ESTs, exonic structure of new isoforms identified with Cap-finder RACE of human retina mRNA and a schema of the new genomic structure containing new TSSs (indicated by red arrows): 4 new cDNAs clones from human retina identify 4 new TSSs for this gene. The transcripts contain two additional exons, a and b, that are alternatively spliced. [file 1471-2164-8-42-S6.pdf]
