# Supplementary material for: Mapping of transcription start sites of human retina expressed genes
Source: BMC Genomics. 2007 Feb 7;8:42. doi: 10.1186/1471-2164-8-42 (PMC1802077; doi:10.1186/1471-2164-8-42)
Supplement: Additional File 7 — Sequence conservation of new first exon. Analysis of sequence conservation of the first new exons of the listed human transcript in comparison with a set of mammals (mouse, dog, cow). Numbers indicate percentage of identity. [file 1471-2164-8-42-S7.doc]

| Sequence conservation of new first exons(Numbers indicate percentage of identity). | | | |
| --- | --- | --- | --- |
| Transcripts | Dog | Mouse | Cow |
| C1orf32 Isoform a | 42 | 45 | 61 |
| C1orf32 Isoform b | 73 | 73 | n.a. |
| CNGA3 | 62 | 51 | 71 |
| DHRS3 | n.a. | 44 | 89 |
| ELOVL5 | 71 | 48 | 63 |
| KIFC3 Isoform a | 50 | 54 | 64 |
| KIFC3 Isoform b | 44 | 43 | 47 |
| RCV1 | 47 | 53 | 58 |
| RDH12 | 45 | 44 | 48 |
| SLC24A2 Isoform a | 56 | 58 | 45 |
| SLC24A2 Isoform c | 77 | 66 | 45 |
